# B Cell Depletion Treatment in Resistant Systemic Sclerosis Interstitial Lung Disease

**DOI:** 10.31138/mjr.33.1.1

**Published:** 2022-03-31

**Authors:** Constantina A. Bounia, Stamatis-Nick C. Liossis

**Affiliations:** 1 Division of Rheumatology, Patras University Hospital, Rion, Patras, Greece,; 2 Departmnnent of Internal Medicine, University of Patras Medical School, Rion, Patras, Greece

**Keywords:** systemic sclerosis, interstitial lung disease, B cell depletion, rituximab, pulmonary involvement

## Abstract

Systemic sclerosis is a systemic, autoimmune disease that in many patients affects not only the skin, but also internal organs, mainly the lung. It is clear that internal organ (ie, lung) involvement determines the prognosis. Therefore, there is an unmet need to introduce novel and more effective treatments capable of halting disease progression and hence improve prognosis. Experimental data over the past decade has accumulated pointing to the B cell as a player in disease pathogenesis. Consequently, a number of controlled and uncontrolled studies have investigated the results of B cell depletion treatment in patients with SSc. The results are preliminary still encouraging for skin as well as for pulmonary involvement. In this review we will analyse and discuss such trials that have currently added B cell depletion as an alternative and promising treatment for resistant interstitial lung disease in scleroderma.

## INTRODUCTION

Systemic sclerosis (SSc) is a chronic, autoimmune and often aggressive disease, mostly because of the progressive involvement of the lungs and other internal organs. Disorders of immune cells and structural damage in blood vessels result in an inflammatory process and a subsequent extensive fibrosis of the skin and internal organs.^1^ Interstitial lung disease (ILD) occurs quite early in SSc, with varying clinical subtypes. Currently, ILD is the principal determinant of disease prognosis and therefore, SSc-ILD is the focus of our review. Screening for SSc-ILD is based mainly on chest high resolution computed tomography images (HRCT) and to a lesser extent to pulmonary function tests (PFTs), which may not be reliable for early detection of the disease. HRCT usually displays 2 patterns: predominantly non-specific interstitial pneumonia (NSIP) and less frequently usual interstitial pneumonia (UIP). Prognosis is considered to be poor if there is progression of ILD in HRCT and decline of PFTs particular of Forced Vital Capacity (FVC) and Diffusing lung capacity for carbon monoxide (DLCO).^2,3^ New treatment options could be based on novel evidence in pathogenetic mechanisms of autoimmunity, vasculopathy, inflammation and fibrosis that lead to disease progression.^1,4,6^ SSc ILD treatment options are few and unable to fight the disease effectively; however, studies are currently testing new drugs.^6–8^ B cell depletion is a treatment option for SSc ILD as assessed by trials during the past 10 years*.* An important number of controlled and uncontrolled studies has been designed to strengthen this assertion.^9,13–28^

Studies in humans with SSc have implicated the semantic role of B cells in the pathogenesis of the disease.^10–11^ Apart from the peripheral blood, B cell infiltrates are apparent in lung tissue from SSc patients as well.^12,13^

## CLINICAL STUDIES OF B CELL DEPLETION

### Controlled studies

Daoussis et al.^9^ from our Department were the first to design and complete a randomized controlled study of rituximab (RTX) in SSc-ILD. The authors evaluated 8 patients receiving RTX as additional therapy to standard treatment, versus 6 patients receiving only standard treatment. All patients who had extensive ILD received RTX at 4 weekly pulses at a dosage of 375mg/m^2^ (“lymphoma scheme”). Patients in the RTX group demonstrated a significant increase of FVC compared to baseline values (mean±S.D.: 68.13 ± 19.69% vs. 75.63 ± 19.73%, at baseline vs 1 year, respectively, *p*=0.0018) and of the DLCO as well (mean ± S.D.: 52.25 ± 20.71% vs. 62 ± 23.21%, at baseline vs. 1 year, respectively, *p*=0.017). On the other hand, the group on standard treatment had no significant changes of FVC and DLCO during the study period. Finally, the chest HRCT scores defining the extent of lung involvement had no worsening at 24 weeks in the RTX group, in contrast to the standard treatment group where a slight worsening was seen; it should be noticed that HRCT scoring was based on a subjective, semi-quantitative system.

Jordan et al.^14^ matched 25 patients treated with RTX with 25 control patients not treated with RTX from the EUSTAR database. The follow-up period was 6 months. RTX-treated patients at 6 months displayed a stable FVC (60.6 ± 2.4% vs 61.3 ± 4.1%; *p*=0.5) and a significantly increased DLCO (41.1 ± 2.8% vs 44.8 ± 2.7%; *p*=0.03) when compared to baseline values. When the authors directly compared the RTX-treated versus the non-RTX-treated groups, significant changes were also revealed in both the percentages of predicted FVC (0.4±4.4 vs −7.7±3.6; *p*=0.02) and the absolute FVC change (0.8±2.2 vs−4.8±1.7; *p*=0.01). On the contrary, DLCO in both groups showed no significant changes (3.7±1.4 vs 6.2±6.2; *p*=0.9).

In a multicentre trial, 7 rheumatology clinics from Greece carried out an open label study of B cell depletion, aiming to evaluate long-term efficacy and safety of RTX in patients with SSc-ILD.^15^ Fifty-one patients received RTX additionally to standard treatment and 33 received standard treatment alone; patients were followed up for a median time of 4 years. During the first 2 years, a significant increase of FVC was shown in the RTX group (mean ± SD of FVC: 80.6 ± 21.2% vs. 86.90 ± 20.56% compared to baseline *p*=0.041). On the contrary, the patients receiving only standard treatment, displayed no significant changes of their FVC. In addition, patients that had been taking RTX for 7 years (n=5) had numerically higher, still statistically not significant FVC increases compared to baseline, (mean ±SD of FVC: 91.60±14.81%, *p*=0.158). However, the patients (n=9) on standard treatment had significant FVC deterioration at 7 years (*p*<0.01). In addition, a significant benefit for the RTX group was displayed in the direct comparison between the two groups (*p* = 0.013). Patients on RTX treatment did not alter DLCO throughout the 7-yr period, while patients on standard treatment alone, displayed a significant reduction of DLCO (*p*=0.004). It is worthwhile to mention that in 6 patients, a temporary cessation of therapy was attempted and was associated with a significant decline of FVC; 3/6 patients only that restarted RTX treatment had a subsequent increase of their FVC. Boonstra M et al.^16^ studied 16 patients with early SSc with a 2-year follow-up; half of them were treated with RTX 1000mg biweekly (“RA scheme”), and the rest of them with placebo treatment. Previous immunosuppressive therapy was allowed for all patients. The study disclosed that FVC and the extent of lung involvement slightly improved with RTX after 2 years, but this difference was non-significant when compared to the placebo group; FVC (placebo: −1.4 vs RTX: +4, *p*=0.65), DLCO (placebo: −2.2, RTX: −6.0, *p*=0.77). Analysis of HRCT lesions according to criteria set by Goh showed a mean change in the percentage of affected lung tissue between baseline and 12 months of −1.6% for the RTX and +2.8% for the placebo treated groups (*p*=0.28). Thiebeau et al.^17^ compared 13 patients with SSc-ILD who received RTX after already having being treated with immunosuppressants with 26 patients with SSc-ILD that did not receive RTX, for a 2-year period. FVC showed no clinically meaningful change at 12 months; 72% at baseline and 85% at month 12 (*p*=0.6), similarly to the DLCO values (40% at baseline vs. 49 % at month 12 [*p* = 0.9]). After 2 years of follow-up, 7 patients treated with RTX improved their FVC with a gain by 12 points. In contrast, 14 patients not treated with RTX worsened their FVC by a loss of −1.5. A direct comparison between the 2 groups revealed a statistically significant improvement of RTX-treated patients (*p*=0.003). DLCO also improved in the RTX-treated group with a gain of 4, while it worsened with a loss of −4.5 in the non-RTX treated group. Once again, the comparison between the 2 treatment groups showed a superiority of RTX-treated patients vs. the non-RTX-treated in the improvement of DLCO (*p*=0.03). Therefore, treatment with RTX disclosed significant improvements in PFTs at 24 months but not earlier. The authors also further analysed a total of 42 patients (35 from the literature and 7 from their own series). This analysis reported an increase of FVC from 71% at baseline to 84% at 12 months (*p*=0.0006) and an increase in DLCO from 58% at baseline to 64% at 12 months (*p*=0.02) in patients treated with RTX.

The EUSTAR study evaluated 146 out of 254 patients with SSc-ILD who received RTX plus standard treatment vs. patients receiving standard treatment alone, for a 2-year period.^18^ FVC remained almost stable during the 2 years in the RTX group (76.3 to 77.7%) and in the standard treatment group as well (79.1 to 80.7%). DLCO also remained stable both in the RTX treated patients (54.4 to 55.5%) and in the standard treated patients (55.6 to 54.7%). Although these patients on RTX group had no significant changes in their lung function, they achieved to reduce the dosage of daily steroids earlier and showed a satisfactory tolerance of the drug. Even though the number of patients was large enough, they had semantic differences in their lung involvement both regarding the extent as well as the chronicity, let alone the protocol of RTX administration that was also different among the participating medical centres. Therefore, a future trial could be designed in order to overcome such confounding heterogeneity that might lead us to superficial and perhaps incorrect conclusions about the effectiveness of B cell depletion.

In a recent open-label, randomised, controlled trial, the authors compared head-to-head RTX vs. monthly cyclophosphamide (CYC) treatment.^19^ Sixty patients with early, diffuse SSc with ILD and anti-Scl70(+), were enrolled to receive RTX or CYC as first-line treatment. Patients in the CYC treatment arm received 500mg/m^2^ CYC IV pulses every 4 weeks for 24 weeks. Patients in the RTX group received RTX as in the RA scheme. The RTX group revealed an improvement of FVC% at the end of 6 months when compared to baseline values (RTX group: 61.3% to 67.5%) while the CYC group did not (CYC: 59.3% to 58.1%). Both the efficacy and the safety demonstrated in this trial, strengthen the statement that RTX may be considered as a first-line therapy versus CYC. However, the current standard-of-care treatment is mycophenolate mofetil (MMF) and not CYC. Another study not completed yet, (RECITAL) also compares RTX versus CYC in the treatment of patients with ILD in connective tissue diseases, not only SSc.^7,20^

An ongoing study evaluates the efficacy and safety of RTX plus MMF in patients with ILDs (EvER-ILD).^7,8,21^ A broad range of patients with non-responding ILD resistant to previous therapy was recruited to receive different treatment schemes; one group received RTX (RA scheme) plus MMF, while the other group received one placebo infusion plus MMF for 6 months. Pulmonary function was evaluated at 6 months and the results are expected with interest. Controlled clinical trials are depicted in **Table 1**. The majority of the studies has noted that B cell depletion treatment was well tolerated. A few gastrointestinal complaints have been reported and to a lesser extent infusion reactions. Our major concern is focused on infections, especially respiratory tract infections.^10,21^ Whether initiating or not initiating B cell depleting therapy we should keep in mind that our patients are highly immunocompromised and have a fibrotic and compromised lung due to SSc.

**Table 1.** Double controlled studies of SSc ILD treatment with RTX.

**Author**	**No. of patients**	**Disease type**	**RTX scheme**	**Follow-up months**	**Outcome**
Daoussis et al. 2010	8	SSc serious ILD RTX add on Tx vs standardTX	Lymphoma scheme	12	Improvement of PFTS compared to controls; steady HRCT lesions
Jordan et al. 2015	25	SSc ILD RTX +stand vs standard Tx	RA scheme	6	Decline in FVC/stable DLCO
Daoussis et al. 2017	51	SscILD RTX add onTx Vs standard Tx	Lymphoma scheme	48	FVC increase/stable DLCO
Boonstra M et al. 2017	16	SscILD	RA scheme	24	Stable PFTs and HRCT lesions
Thiebeau et al. 2018	13	Progressive Ssc ILD	RA scheme	24	No difference compared to control/improvement of PFTs in RTX group
Sircar et al 2018	25	SSc ILD RTXvs CYC	RA scheme	6	Improved FVC compared to CYC
M Elhai, M Boubaya et al. 2019 EUSTAR	146 patients	SSc ILD	RA scheme	24	Stable PFTs

### Uncontrolled Clinical Studies

Lafyatis et al.^13^ treated 15 patients with early SSc with RTX only (RA scheme) without concomitant disease modifying anti-rheumatic (DMARD) therapy; they did not find a clear beneficial effect on skin fibrosis and pulmonary function at 6 and 12 months of follow-up. It should be stressed though that patients with severe lung disease were excluded from this trial. Therefore, it is not surprising that the average FVC and DLCO showed no significant differences at 6-months (92.7% and 77.9 % predicted, respectively) compared to the baseline (89.2% and 79.7 % predicted, respectively) because they were practically normal before enrolment. The same stands for little or no progression of pulmonary disease depicted at HRCT. Since patients included in this study had near-normal respiratory function, no conclusion can be made upon the above results for evaluating the effects of B cell depletion treatment.

Daoussis et al.^23^ showed a beneficial effect of RTX (lymphoma scheme) when the follow-up of our initial cohort of 8 patients that were previously analysed and published at 1 year was extended to 2 years of treatment. FVC values displayed a remarkable increase at 2 years (mean±SEM: 77.13±7.13% vs 68.13±6.96%, respectively, *p<*0.0001) as did the DLCO values (mean±SEM: 63.13±7.65% vs 52.25±7.32%, respectively, *p*<0.001). Moreover, HRCT analysis depicted lesser ground glass lesions in 5/8 patients. The results are considered to be encouraging, even though the numbers of patients enrolled were small and RTX was administered concurrently with other immunosuppressing drugs (for instance MMF).

A promising open-label study by Smith et al.^24^ in patients with early diffuse SSc who received RTX for a 2-year period, showed a statistically significant overall decrease of FVC, with a mean FVC 92.8% at baseline versus 84.7% at 24 months (*p*=0.047). This was not in agreement with any clinical worsening. DLCO remained stable over the 2 years. A reduction of >10% yearly of a PFT parameter like FVC is arbitrary and of uncertain clinical significance, whatsoever.

Bosello et al.^25^ administered RTX (RA scheme) in 20 patients with early (< 3 years disease duration) and extensive SSc-ILD. During the follow-up period of 2 years, the evaluation of PFTs every 6 months and of chest HRCT every 12 months indicated a stability of FVC, DLCO and chest HRCT lesions over time. However, a marked heterogeneity was recorded for follow-up duration and for the number of RTX cycles administered. When analysed separately, the patients with restrictive disease (n=6) in PFTs (representing perhaps the most interesting patient subset) had an increase of their FVC but insignificant changes of their DLCO and HRCT scores during the first year of RTX treatment. In contrast, patients without restrictive disease in PFTs (n=8) displayed no increases of the above parameters throughout the study.

In a retrospective analysis, Sharp et al.^26^ studied 24 patients with resistant, non-responsive to previous treatment SSc lung disease. B cell depletion resulted in stabilization of FVC, DLCO and chest HRCT parameters of these patients. Lepri et al.^14^ analysed retrospectively patients with ILD of different aetiologies, including 23 patients with SSc-ILD. Patients were treated with RTX plus other DMARDs and were followed-up for 2 years. In patients with SSc-ILD, FVC changed from 81.0% at baseline to 89.0% at 1 year (*p*=0.1) and 74.5 at 2 years (*p*=0.07). DLCO changes remained non-significant throughout*.* The results of this uncontrolled trial are confounded by the retrospective nature of the study and by the unclear RTX-dosing regimen.

Vilela et al.^27^ described 10 patients with diffuse SSc that received RTX (RA scheme) and were evaluated 6 months later. There were no significant changes in PFTs in all patients, neither in the subgroup with early disease (duration less than 4 years and refractory to standard treatment), nor in the subgroup with worsening, progressing pulmonary function. Sari et al.^28^ claimed a beneficial effect of RTX therapy in 14 patients with SSc and chronic extensive lung involvement resistant to previous therapy. Although encouraging, the results are not comparable between patients because they received different numbers of cycles and different dosages of RTX. Uncontrolled clinical trials are depicted in **Table 2**.

**Table 2.** Uncontrolled studies of SSc-ILD treatment with RTX.

**Author**	**No. of patients**	**Disease**	**RTX scheme**	**Follow-up months**	**Outcome**
Lafyatis et al. 2009	15	Early SSc ILD RTX add on standard Tx not progressive ILD	RA scheme	24	Stable PFTs no ILD progression
Daoussis et al. 2012	8	SSc ILD RTX add on standard Tx	Lymphoma scheme	24	Increase of FVC, DLCO, decrease ground class
Bosello et al. 2015	20	Early serious ILD SSc	RA scheme	24	Stable PFTs
Sharp et al. 2016	24	Resistant SSc ILD to previous Tx	RA scheme	24	Stable PFTs
Sari et al. 2017	14	Resistant SSc ILD	RA scheme	6	Stable PFTs

### Meta-analysis

A recent systematic review and meta-analysis of Goswami et al.^29^ evaluated the efficacy of RTX on the pulmonary function parameters of patients with SSc-ILD. Twenty studies were included in this meta-analysis and specifically 2 randomized controlled trials, 6 prospective studies, 5 retrospective studies and 7 conference abstracts. RTX was reported to provoke increase of FVC by 4.49% (95% CI 0.25, 8.73) and increase of DLCO by 3.47% (95% CI 0.99, 5.96) at 6 months. In addition, FVC was increased 7.03% (95% CI 4.37, 9.7) and DLCO 4.08% (95% CI 1.51, 6.65) after one year treatment with RTX. Only two studies comparing RTX administration to other immunosuppressants showed improvement of FVC 1.03% (95% CI 0.11, 1.94) greater than controls at six months but not at 12 months. The change of DLCO was similar at both groups (RTX-treated and controls) at both time points of 6 and 12 months. The use of sensitivity analysis because of the heterogeneity of the studies included in the meta-analysis ended up with almost similar results on lung function tests. The authors acknowledge another limitation of this meta-analysis such as the different disease traits, RTX scheme and evaluation of PFTs at standard time points of 6 and 12 months.

## CONCLUSION

SSc-ILD is a challenging, progressive, and in some patients, a life-threatening disease. Therefore, an aggressive and effective treatment with an acceptable safety profile seems imperative to slow down at least disease progression.

Apart from the current standard-of-care treatment with MMF, B cell depletion appears to be beneficial and acceptably safe for scleroderma lung disease: this is the reason why it is widely used, at least in tertiary-care centres. Moreover the 2 different treatment approaches can be combined successfully. A good number of open-label studies verify the above statement. Both controlled and uncontrolled studies of using treatment with RTX are encouraging in their vast majority. Based on data presented in this review, patients with SSc-ILD, with either early-or long-standing disease that is resistant to previous therapy, may respond successfully to B cell depletion treatment. In addition, imaging studies may also depict improvements following RTX treatment, despite their semi-quantitative approach. Repeated cycles of B cell depletion leading to a long-term treatment is considered to be a better approach in contrast to a single infusion. Published trials present multifactorial heterogeneity. Differences have been identified in disease duration, in co-treatments and in the extent of lung involvement. The randomly defined values for PFT parameters (such as the FVC and DLCO) and the lack of a widely acceptable and reliable scoring system for HRCT lesions make it difficult to attain a clear benefit from B cell depletion treatment in patients with SSc-ILD. A well-designed, well-stan-dardised, large enough study is needed to overcome such limitations. Employing B cell depletion as a first-line treatment is also another question to be addressed. Despite all the above limitations and downsides, one should keep in mind that the vast majority of studies published so far have supported that B cell depletion as a therapeutic approach induces a significant improvement in patients with SSc-ILD.
